# Effects of early maternal separation on the expression levels of hippocampal and prefrontal cortex genes and pathways in lactating piglets

**DOI:** 10.3389/fnmol.2023.1243296

**Published:** 2023-08-14

**Authors:** Sitong Zhou, Yue Yang, Zheng Cheng, Mengyao Wu, Qi Han, Wenzhong Zhao, Honggui Liu

**Affiliations:** ^1^College of Animal Science and Technology, Northeast Agricultural University, Harbin, Heilongjiang, China; ^2^Institute of New Rural Development, Harbin, Heilongjiang, China; ^3^Key Laboratory of Swine Facilities Engineering, Ministry of Agriculture and Rural Affairs, Harbin, Heilongjiang, China

**Keywords:** piglets, maternal separation, hippocampus, prefrontal cortex, neurodevelopment, cognitive

## Abstract

**Introduction:**

In actual production, due to increased litter size when raising pigs, the management of piglets by split-suckling leads to intermittent neonatal maternal separation (MS). Early lactation is a critical period for the cognitive development of the brain of newborn piglets, and we hypothesized that intermittent MS may affect piglets’ neurodevelopment and cognitive ability.

**Methods:**

To determine the effects of the MS, we selected hippocampal and prefrontal cortex (PFC) tissues from piglets for the detection of neurodevelopmental or cognitive related indicators, the control group (Con group, *n* = 6) was established with no MS and an experimental group (MS group, *n* = 6) was established with MS for 6 h/day. Piglets in the MS group were milk-supplemented during the separation period and all piglets in both treatment groups were weaned at postnatal day (PND) 35. On PND 35, three male piglets from each group were sacrificed for hippocampus and PFC samples used for reference transcriptome sequencing. Following bioinformatics analysis, Gene ontology (GO) enrichment, Kyoto encyclopedia of genes and genomes (KEGG) enrichment analysis, and candidate gene screening and pathway were performed for differentially expressed genes.

**Results:**

The results showed that a total of 1,632 differential genes were identified in the hippocampus of the MS group, including 1,077 up-regulated differential genes, 555 down-regulated differential genes, and 655 significant GO entries. Analysis of the PFC of the MS group revealed 349 up-regulated genes, 151 down-regulated differential genes, and 584 significant GO entries. Genes associated with neurodevelopment were screened for large fold differences in the hippocampus, and genes associated with cognition were screened for large fold differences in the PFC. Quantitative real-time PCR (qRT-PCR) was used to verify the sequencing data. Western blot (WB) experiments revealed that MS inhibited the neurodevelopment-related WNT signaling pathway in the hippocampus and the cognitive-related PI3K-AKT signaling pathway in the PFC.

**Discussion:**

Taken together, these findings suggest that intermittent MS may affect some cognitive functions in piglets by damaging hippocampal and PFC genes or pathways.

## 1. Introduction

With advances in pig production, feeding management technology, and the deepening of genetic breeding, the number of piglets born per litter has gradually risen. Often piglets cannot grab the teat of the sow, resulting in their malnutrition or even death. Production methods applied to solve this problem include artificial nursing, split-suckling (when the number of piglets in a sow’s litter is more than the number of effective teats, at the end of labor the litter is divided into two parts for lactation), and cross-fostering (whereby some piglets are fostered to another sow that has recently give birth and can produce enough milk). However, artificial nursing and split-suckling of piglets can lead to a degree of neonatal maternal separation (MS), meaning termination of the continuity of the mother-offspring relationship after its establishment. Stress induced by MS has important implications for neurodevelopment and disease risk in the offspring (Loewy et al., 2019). In previous experiments we found that intermittent neonatal MS led to both physiological stress and increased aggressive behavior in piglets (Cheng et al., 2023), and that this largely associated with impaired cognitive ability (Yu et al., 2019). The brain is crucial for the regulation of the bodily behavior, and the suckling period is a critical period for the brain development of newborn piglets. Therefore this study investigated whether the stress caused by the neonatal MS has an impact on the neurological development and cognitive function of the piglet brain.

In recent years, MS has become a focal area in research on animal stress. Early studies have shown that MS causes locomotion deficits in CD1 mice at puberty and abnormal activation of microglia in the prefrontal cortex (PFC) and hippocampus, resulting in neuroinflammation and leading to emotional disorders (Gracia-Rubio et al., 2016). Using a newly developed Multiple Animal Positioning System, Endo et al. (2021) found that repeated daily separation of mothers, an early life stressor, caused C57BL/6J mice to exhibit lower activity levels and altered social behavior, showing longer social distances. A study by O’Mahony et al. (2011) showed that neonatal MS significantly reduced stressor-induced corticosterone secretion in mice on PND 14, though impairment of the hippocampal negative feedback mechanisms to the hypothalamic–pituitary–adrenal (HPA) axis. It was also been concluded that mice that underwent MS in the first 3 weeks of life could develop anxiety in their adulthood or might have decreased cognitive ability that could cause memory impairment in adulthood (Banqueri et al., 2017). Similar findings were found for primates, MS also led to HPA axis dysfunction and behavioral abnormalities in rhesus monkeys (Feng et al., 2011). It has been demonstrated in many animal models that MS causes dysfunction of the HPA axis by modifying glucocorticoid release (Sanchez, 2006). The above studies concluded that early-life stress caused by neonatal MS can damage the development and function of the individual’s brain, and that the regulatory mechanisms are diverse. However, there are few studies have considered the effects of MS stress on piglets.

Through dissection it has been determined that the hippocampus is phylogenetically one of the oldest regions of the brain and an essential component of the brain’s limbic system, located deep within the medial part of the brain, with a unique shape and cellular structure, and that hippocampal neurodevelopment is closely related to the degree of cognition in the organism. The PFC is the cerebral cortex covering the anterior frontal lobe and is thought to underlie the rich and complex nature of animal cognition (Ma et al., 2022). The PFC coordinates brain activity well owing to the extensive connection of different brain areas. Negative stimuli from the outside world can produce stress in animals, in turn causing impaired hippocampal and PFC function. For example, rat pups exposed to daily 3-h periods of MS stress during a critical period of hippocampal development disrupted hippocampal cell structure, with the resulting morphological changes likely contributing to learning deficits and stress hyperresponsiveness (Huot et al., 2002). Fabricius et al. (2008) found a significant reduction in neurons in the dentate gyrus neurons in mice that underwent MS on postnatal day (PND) 9. Psychological stress generated by external stimuli can cause a neuroinflammatory response in the hippocampus by activating microglia, increasing microglia activity, and releasing inflammatory factors (Calcia et al., 2016). In sows, repeated stress from long-lasting tethering leads to an overactive neuroendocrine system in pigs, reducing levels of brain-derived neurotrophic factor (BDNF) protein (a key mediator of activity-dependent neuronal connectivity and synaptic plasticity in the brain) in the dorsal hippocampus and frontal cortex (De et al., 2012). In rats, it has been suggested that neonatal MS for 24 h can cause a reduction in PFC thickness and a decrease in the number of mature neurons in the PFC in rats (Crombie et al., 2021). MS stress in juvenile rhesus monkeys led to activation of the right dorsolateral PFC and the right ventral temporal/occipital lobes, while activity in the left dorsolateral PFC was reduced (Rilling et al., 2001). Research using microarrays and quantitative real-time PCR (RT-PCR), found that the stress caused by early weaning and social isolation affected the expression of genes involved in the regulation of neuronal function and development in the PFC of piglets (Poletto et al., 2006). However, there have been few studies on the effects of stress from neonatal MS on the hippocampus and PFC of piglets, and the molecular mechanisms remain unclear.

The domestic pig is a traditional animal model. Because of high similarities between pigs and humans in aspects of their anatomy, physiology, biochemistry, immunology and genomes, the pig model is widely used in laboratory and clinical research (Lunney et al., 2021). Compared with primates and other domestic animal models, pigs have several attributes that are advantageous, including a short reproductive time and large litter size. Since some aspects of pig production inevitably cause neonatal MS, the neonatal MS model has a theoretical basis and is extremely important for studying the molecular mechanisms of the effects of negative stimuli on animals and the trauma to animals. However, there have been few studies related to the effects of neonatal MS separation in piglets, including the molecular mechanisms. Transcriptomics technologies are revolutionizing our understanding of transcriptomes. Transcriptome sequencing analysis is an indispensable technical tool for biological and medical research. It enables researchers to uncover the molecular mechanisms underlying biological phenomena or disease occurrence. This technique allows for the quantification of the full spectrum of genes and the identification of differentially expressed genes (DEGs) and related molecular pathways. Up to now, few researchers have analyzed the mechanisms by which MS affects brain development and function in pigs at the molecular level. Additionally, there are few sufficiently reliable neonatal MS models in pigs. Therefore, the aim of this study was to develop a model of neonatal MS in pigs to investigate the mechanisms underlying the stress response. The hippocampus and PFC were selected as experimental tissues, and the use of transcriptomic, quantitative real-time PCR (qRT-PCR), and protein immunoblotting techniques will provide information to better assess the mechanisms of neonatal MS stress on the hippocampus and PFC of piglets, as well as provide a reference for clinical veterinary and animal production management and farm-animal health and welfare.

## 2. Materials and methods

### 2.1. Experimental animals

The trial site was the experimental demonstration base of Acheng, Northeastern Agricultural University. This experiment involved 12 litters of crossbred piglets from healthy Du Min (Duroc × Min-pig) sows of similar body condition. The same bloodline was used to ensure the same genetic background of the piglets used in testing. The mating boars were American Large White pigs. The sows were transferred to farrowing pens 1 week before farrowing. The experiment consisted of two treatment groups, each with six replicates (six litters), eight piglets per litter (random selection). In the control (Con) group (*n* = 6), piglets were fed at birth after colostrum and immunization until weaning on postnatal day (PND) 35. In the maternal separation (MS) group (*n* = 6), sows were induced into a new pen with food from 8:00–11:00 to 13:00–16:00 daily (6 h in total) for intermittent neonatal MS starting from PND 7 until weaning on day 35. Sows and their piglets were housed in loose farrowing and nursery pens (measuring 5.2 m × 1.6 m × 1.1 m) with feed troughs and waterspouts at the front of the pens, and the floor type is slatted floor. The temperature and humidity of the pens were maintained at a comfortable and constant level. The environment was controlled using an automatic ventilation system. The piglet rest area had an insulation heating device and an average temperature of 26.2it an average humidity of 68.3%, natural lighting, and good ventilation. The sows and piglets were fed and raised in strict accordance with the production process requirements. The sows were fed daily at 6:00, 11:00, and 17:00, while the piglets had free access to food and water. Piglets received iron supplements at PND3 and were fed creep feed from PND7. The compositions of the lactating sows feed, piglet creep feed during the experiment were presented in the Supplementary Table 1. The pens were kept clean and hygienic and were regularly disinfected (the pens were not disinfected during the whole lactation period). The piglets were vaccinated (Immunization of 1–3 day piglets with pseudorabies gE gene deletion vaccine by intranasal vaccination, 14 day vaccination against circovirus, vaccination against swine fever at 28 day, second immunization for pseudorabies gene deletion at 35 day). To reduce stress, the piglets in this study were not tail docked or castrated. At PND35, three male piglets in good condition were selected for slaughter in each of two groups (Con group, *n* = 6 litters; MS group, *n* = 6 litters). Afterward, the brain tissue was quickly removed and the 1 × 1 cm^3^ size hippocampal and PFC brain region were collected in RNAase-free EP tubes and stored at −80°C for subsequent reference transcriptomic analysis, quantitative real-time fluorescence PCR (qRT-PCR) and western blot detection.

Hippocampal tissue removal procedure: the skin was cut in the middle parallel to the direction of the nasal bone, and the skin was peeled from the nasal bone to the skull to expose the skull. Then, in the perpendicular and parallel directions of the nasal bone, the nasal bone was split, and then the bones on both sides were split to expose the brain tissue. It was transferred to a petri dish and placed in PBS solution at a concentration appropriate to the osmotic pressure of the tissue. The brain tissue was cut in half dorsally with a clean scalpel. The tip of the scalpel blade was fixed near the cerebellum where it intersected the cerebral cortex, and the tip of the other blade was placed at the same junction to peel the cerebral cortex hemisphere to one side. The hippocampus (large “C” shape) is exposed. The hippocampus is “spooned” or “rolled” to one side with the tip of the forceps.

Prefrontal cortex tissue removal procedure: the process of removing brain tissue is the same as in the hippocampus. The PFC is located in the superficial layer of the anterior part of the brain tissue.

### 2.2. Transcriptomics profiling technique

Total RNA was isolated and purified from total samples using TRIzol (Invitrogen, USA), followed by quality control of the quantity and purity of total RNA using NanoDrop ND-1000 (NanoDrop, USA). The fragmented RNA was synthesized into cDNA by reverse transcriptase (Invitrogen SuperScript™ II Reverse Transcriptase, No. 1896649, CA, USA), which was then used to form a cDNA library with a fragment size of 300 bp ± 50 bp. Transcriptome profiling steps were carried out by LianChuan-biotechnology Co., Ltd. (Hangzhou, China). The sequencing data were screened and sorted using a Hiseq™ sequencer, and genes with the |log_2_ (Fold Change)| greater than 1.5 and *P-*value less than 0.05 were defined as differential genes. Finally, significant difference analysis was performed using the R package DESeq2, GO and KEGG enrichment analyses were performed (Wang et al., 2021). For the assessment of the quality of the RNA-seq library results metrics, we used the RSeQC software (version 2.6.4) to pre-process the raw data, including the removal of sequencing junctions (introduced during the library construction process) and low-quality sequencing data. Valid data were obtained after filtering out substandard sequences using Cutadapt) before proceeding to the next step of analysis. The specific data preprocessing steps are as follows: (1) Remove reads with adaptors; (2) Removal of reads containing N (N indicates that base information cannot be determined) in a proportion greater than 5%; (3) Removal of low-quality reads (the number of bases with a quality value Q ≤ 10 accounted for more than 20% of the entire read); (4) Statistics of raw sequencing volume, effective sequencing volume, Q20, Q30, GC content, and comprehensive evaluation.

### 2.3. qRT-PCR verification of DEGs

Total RNA was extracted from the hippocampal and PFC of piglets with TRIzol reagent (Invitrogen, China) according to the instructions of the RNA Extraction Kit (Beijing Mei5 Biotechnology Co., Ltd., China). The concentration and purity of total RNA were determined by using Quick Drop™, an ultra-micro-analyzer (Valley Molecular, Inc., USA). The mRNA was reverse transcribed according to the reaction system (20 μL) of the Reverse Transcription Kit (Beijing Mei5 Biotechnology Co., Ltd., China). Using Primer Premier 5.0 design specific primers for the screened differential gene and the internal reference gene β-actin based on the gene sequences published in GenBank. Primers were synthesized by Sangon Biotech Co., Ltd. (Shanghai, China). The specific primer sequences of the screened differential and internal reference genes β-actin were shown in Table 1. qRT-PCR was performed using the LightCycler^®^96 (Roche, Switzerland) for quantitative real-time PCR (qRT-PCR). The relative expression of mRNA of each gene was calculated using 2^–ΔΔCT^.

**TABLE 1:** Gene-specific primers used in qRT-PCR.

Gene	Forward primer (Five’-Three’)	Reverse primer (Five’-Three’)
PPP3CA	GCCAACACTCGCTACCTCTTCTTAG	AGCCCACAAGTACAGCACACATTC
RNF112	CTGCTGGGGAAGGAGGGGAAG	GAGGCGGCGAGGATCTGGTAG
DAB1	CCACGCCATCGACCAACTCAC	CTCTTCGCTCTTGCTGGGACTTTC
NEUROD1	GGAAGAGGAGGAGGAAGAGGAAGAG	GCGTTGGCTTTCATTCGTCTCAG
RFX3	CTCCTTCCACCTGATCCGTCTACT	CCAAACTCACCCATGACCGCTATG
RSPO2	CCATGTCCAACCATTGCTGAATCC	CTGTGTTGCTCCTGGGCTCTATC
FZD5	AGCCACATCCACTACGAGACAAC	CCAAGAACCAGGTGAGCGACAG
WNT2	CTGTTCCTGTGACCCGAAGAAGAAG	GCTCTGGCGTCCTTTCCTTTCC
WNT5A	CTTCAACTCGCCCACCACACAG	ACAGCCGTCCATGCCCTCAG
WNT10B	GTCTCCTGTTCCTGGCGTTGTG	GCCTAACTGCCGCTTGCTCAG
LRP5	ACGCCAAGACAGACAAGATAGAGG	GCCAGTCGGTCCAGTAGATGAAG
LRP6	ATGCGAGAAGGGAAGATGGG	CTCTCAGGAGCACGCAGAAAC
β-Catenin	CCATCTGTGCTCTCCGTCATCTG	GGTAGTCCGTAGTGAAGGCGAAC
AXIN2	ATGAGGAGGACCCGCAGACC	GTGGTGGTGGTGGTGGTGATG
GSK-3β	GCCCAGAACCACCTCCTTTGC	TCACCTTGCTGCCATCCTTGTC
APC	TCAGCAAACGCAGGAAACAGATTC	TGGAACTTCGCTCACAGACTCTTC
MC4R	GCTGTGGCTGATATGCTGGTGAG	GAAACTCTGTGCGTCCGTGTCC
GRP	CTGGGCGGTGGGACACTTAATG	CAGCAAATTCCTTGTGGCATCTTCC
CYFIP2	CACATCCGCTTCATCTCCGAACTC	GGTACTCCTCGTCCGACTTCTGG
NMB	GCCTCCTGTTCTTCACTCTGCTC	GCTCCAGACTCTTCTTGCCCATG
CPEB1	CCTGGTCCTTTCTTCTGTCGTGATC	CTGGCATCTCGGTTCTTCTGGTTC
ADCYAP1	AAGAGATGTCGCCCACGGGATC	TCCGAGTCATCTTCCGCTCCTC
MAG	ATTGCCATCGTCTGCTACATCACC	GGAAGTCGCTGCTGAACAGGAC
PLP1	GGCTAGGACATCCCGACAAGTTTG	GGAAGGCAATAGACTGGCAAGTGG
CPEB4	TCGGCTAATAACGGTGCTCTGTTG	TGCTGATGCTGGCTGTGATGATG
PI3K	ACGGCAATGTGGAGCAGATGAAG	TGGTAGAGCAGGAGGAAGTGGTC
AKT	TCAAGAACGACGGCACCTTCATC	CGCCACGGAGAAGTTGTTGAGG
β-actin	GGCACCACACCTTCTACAACGAG	TCATCTTCTCACGGTTGGCTTTGG

### 2.4. Western blot detection

To determine the protein levels, we extracted total proteins from hippocampal as well as prefrontal tissues using Western and immunoprecipitation (IP) lysis buffer (P0013, Biosharp, China) containing protease inhibitors and phosphatase inhibitors (Beyotime, China). Protein concentrations were determined according to the BCA Protein Assay Kit (Beyotime, China) instructions using the sequence. Electrophoresis of 20 μg of lysate was performed through 10 or 12% SDS-PAGE gels (Solarbio, China). The proteins were then transferred to polyvinylidene fluoride (PVDF) membranes (Cytiva, Marlborough, MA, USA). The membrane was blocked with 5% BSA for 1 h at room temperature, incubated with primary antibody overnight at 4°C, and then incubated with secondary antibody for rabbit IgG (1:5,000, Santa Cruz, CA, USA) or secondary antibody for mouse IgG (1:5,000, Proteintech, China) for 1 h at room temperature. The primary antibodies used in this experiment were Roof Plate-Specific Spondin-2 (RSPO2, 1:1,000), Frizzled Class Receptor 5 (FZD5, 1:1,000), β-catenin (1:1,000), Axis inhibition protein 2 (AXIN2, 1:1,000), Glycogen Synthase Kinase-3β (GSK3-β, 1:1,000), Adenomatous polyposis coli (APC, 1:1,000), WNT Family Member 10B (WNT10B, 1. 1,000), WNT Family Member 2 (WNT2, 1:1,000), WNT Family Member 5A (WNT5A, 1:1,000), LDL Receptor Related Protein 5 (LRP5, 1:1,000), LDL Receptor Related Protein 6 (LRP6, 1:1,000), Phosphatidylinositol-3-kinase (PI3K, 1: 1,000), Protein kinase B (AKT, 1: 1,000), Phospho-Phosphatidylinositol-3-kinase (p-PI3K, 1: 500), Phospho-Protein kinase B (P-AKT, 1: 500), Phospho-Glycogen Synthase Kinase-3β (P-GSK3-β, 1,500), and β-actin (1:5,000) was used as reference for analysis. Syngene G:BOX Chemi XX9 imager (Syngene, Cambridge, UK) was used to detect protein signals using enhanced chemiluminescence (ECL) reagents (Beyotime, China). Finally ImageJ software (National Institutes of Health, Bethesda, Maryland) was used to quantify the relative density of the bands.

### 2.5. Statistical analysis

Statistical analysis of all data was performed using GraphPad Prism software (version 8.0, GraphPad Software Inc., San Diego, CA, USA) and used SPSS 22.0 for data analysis. First, testing data were for normal distribution by Shapiro–Wilk test method, and the Levene test was used for homogeneity of variance test, to ensure that all data from the same treatment group conform to the normal distribution, the homogeneity of variance > 0.05. Quantitative data were expressed as mean ± standard deviation (SD). To analyze the data, the one-way analysis of variance (ANOVA) and *post-hoc* test by Turkey were performed. *P* < 0.05 was considered statistically significant.

## 3. Results

### 3.1. Differential gene expression and analysis

**FIGURE 1:**
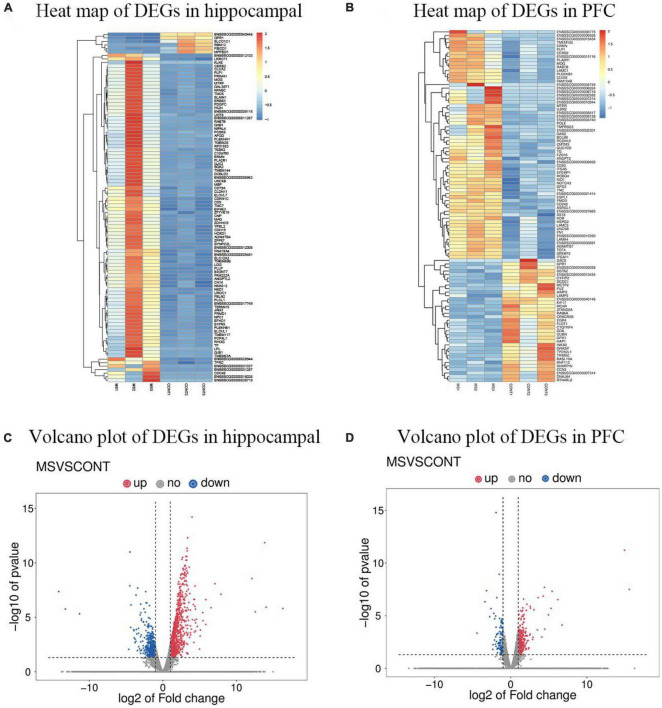
DEGs profiling using high-throughput transcriptomics sequencing technology (*n* = 6). **(A)** Heat map of DEGs in hippocampal of MS group vs. Con group. **(B)** Heat map of DEGs in PFC of MS group vs. Con group. “ 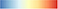 ” Image represents the expression level of differentially expressed genes upregulated from left to right. **(C)** Volcano plot of DEGs in hippocampal for MS group vs. Con group. **(D)** Volcano plot of DEGs in PFC for MS group vs. Con group. Red indicates upregulation, blue indicates downregulation.

To investigate the changes in the genetic levels of hippocampus and PFC in MS piglets, transcriptome sequencing was used to determine the genetic levels of hippocampus and PFC. The differential genes were classified into Up-regulated Gene and Down-regulated Gene according to the relative levels of gene expression between the two groups of samples. Comparing the gene expression of intermittent MS piglets and normal reared piglets, a total of 1,632 differential genes were identified in the hippocampus, including 1,077 Up-regulated differential genes and 555 Down-regulated differential genes, and a total of 500 differential genes were identified in the PFC, including 349 Up-regulated differential genes and 151 Down-regulated differential genes (Figure 1).

### 3.2. Differential gene GO enrichment analysis

**FIGURE 2:**
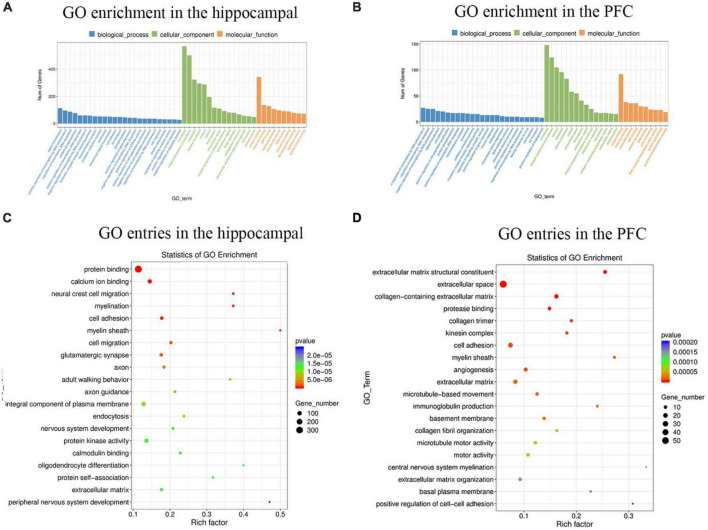
The histogram and scatter plot of GO enrichment analysis in the hippocampal and PFC. **(A)** Statistical analysis of GO enrichment in the hippocampal. **(B)** Statistical analysis of GO enrichment in the PFC. Blue represents biological processes, green represents cellular components, and orange represents molecular functions. The 25 biological process entries, 15 cellular component entries, and 10 molecular function entries with the most significant differences in the hippocampus and PFC, respectively, were selected for analysis. The specific title of the Go entry see Supplementary Table 2. **(C)** GO entries in the hippocampal. **(D)** GO entries in the PFC. The horizontal coordinate indicates the enrichment level of each GO entry. The size of the dot indicates the number of genes enriched to this entry. The color of the dots indicates different *p*-values. 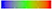 represents a gradual increase in the *p*-value.

To better understand the gene functions and gene products involved in MS in hippocampus and PFC, the GO (Gene ontology) database was used to analyze DEGs (differentially expressed genes) in hippocampus and PFC. The results of GO enrichment analysis of differentially expressed genes. GO has three ontologies, biological process, cellular component and molecular function. There were 5,612 GO entries (655 significant) for differentially expressed genes in the hippocampus and 2,537 GO entries (584 significant) for differentially expressed genes in the PFC. In the hippocampus (Figure 2A), the top 5 GO entries in the biological process were: signal transduction, positive regulation of transcription by RNA polymerase II, regulation of transcription, DNA-templated, protein phosphorylation, positive regulation of transcription, DNA-templated. The first five GO entries in the cellular component are: membrance, integral component of membrane, cytoplasm, plasma membrane, nucleus. And the top 5 GO entries in molecular function are: protein binding, metal ion binding, ATP binding, nucleotide binding, transferase activity. In the PFC (Figure 2B), the first five GO entries in the biological process are: positive regulation of transcription by RNA polymerase II, negative regulation of transcription by RNA polymerase II, regulation of transcription, DNA-templated, signal transduction, cell adhesion. The first five GO entries in cellular component are: membrane, integral component of membrane, nucleus, cytoplasm, plasma membrane The first five GO entries in molecular function are: protein binding, metal ion binding, nucleotide binding, ATP binding, and zinc ion binding.

The top 20 GO entries with the most significant enrichment were selected for display, and the results of the enrichment bubble chart display were shown in Figures 2C, D. Among the top 20 GO entries in the hippocampus, protein binding genes was the most abundant and had the most significant differences, and myelin sheath had the highest number of enrichment factors. Similarly, it can be concluded that in the PFC, extracellular space had the highest number of genes, extracellular matrix structural constituent had the most significant differences, and central nervous system myelination had the highest number of enrichment factors.

### 3.3. Validation of differential gene screening and transcriptome sequencing results

**FIGURE 3:**
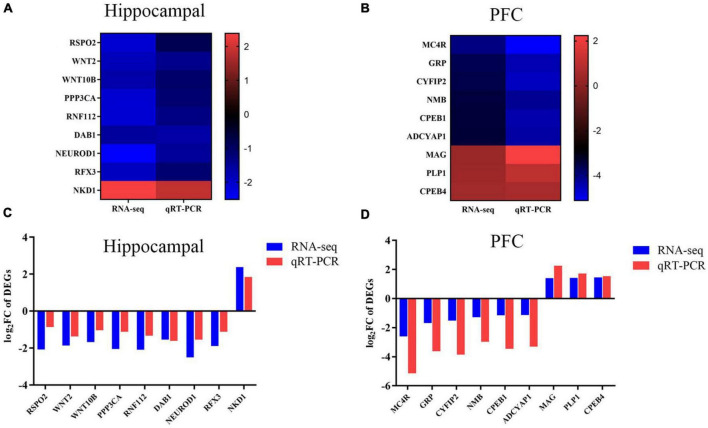
Trends in the expression levels of DEGs in hippocampus and PFC. **(A)** Genes in the hippocampus. Heat map shows the relative expression levels of selected genes related to neurodevelopment. The color bars in the heat map show the trends of differentially expressed genes. **(B)** Genes in the PFC. Heat map shows the relative expression levels of selected genes related to cognitive ability. The color bars in the heat map shows the trend of differentially expressed genes. **(C)** Genes in the hippocampus. qRT-PCR validation of DEGs in the hippocampus between CON and MS groups. **(D)** Genes in the PFC. qRT-PCR validation of DEG in PFC between CON and MS groups. The log_2_FC is used here to be consistent with the representation of the transcriptome results. Each value is expressed as mean ± SD (*n* = 6). Relative expression levels of mRNA were calculated based on the 2^–ΔΔCt^ method.

To ensure the authority of the results of transcriptomic analysis, we verified the trends of the genes selected in each group by qRT-PCR. Genes related to neurodevelopment were screened in the hippocampus and genes related to cognitive memory were screened in the prefrontal by the literature and the results of this experiment, |log_2_FC| > 1 and *P* < 0.05. These candidate genes were validated using qRT-PCR. A comparative analysis of transcriptome sequencing results and qRT-PCR results were shown in Figure 3. In the hippocampus, compared with controls, MS stress caused WNT2, WNT10B, RSPO2, PPP3CA, RNF112, DAB1, NEUROD1, and RFX3 gene expression levels were downregulated and NKD1 expression levels was upregulated. In the PFC, MC4R, GRP, CYFIP2, NMB, CPEB1, and ADCYAP1 were significantly down-regulated and MAG, PLP1, and CPEB4 were significantly up-regulated in piglets of the MS group compared with the control group. qRT-PCR were consistent with the trend of transcriptome sequencing results, indicating that the transcriptomic analysis had better confidence.

### 3.4. Enrichment analysis of differentially expressed gene KEGG

**FIGURE 4:**
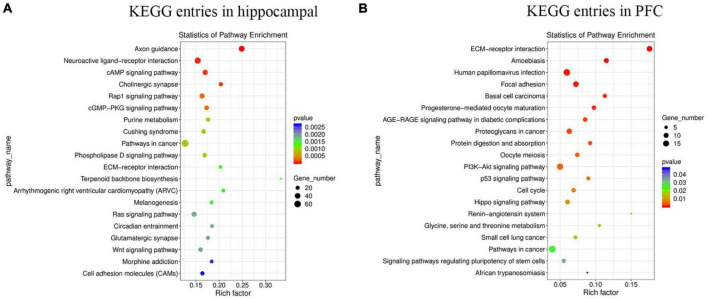
The enrichment of KEGG terms in hippocampus and PFC was analyzed by bubble plots, and the top 20 KEGG entries with the most significant enrichment were selected for display. The horizontal coordinates indicate the degree of KEGG entry enrichment (Rich factor), and the size of the dots indicates the number of differential genes enriched in a particular KEGG pathway; the color of the dots indicates different *p*-values. **(A)** KEGG entries in hippocampus. **(B)** KEGG entries in PFC.

Kyoto encyclopedia of genes and genomes helps to study gene and expression information as a whole network. To better understand whether the differential genes involved in MS in hippocampus and PFC occur significantly differently in a given pathway, the samples were analyzed using the KEGG database. The results of the KEGG enrichment analysis were plotted as bubble plots (Figure 4), which present the top 20 pathways with the lowest significant *P*-values in hippocampus and PFC. Among the top 20 pathways in the hippocampus, the Axon guidance pathway had the most significant differences and the highest number of enriched factors, and the Pathways in cancer pathway had the highest number of genes. Among the top 20 pathways in the prefrontal lobe, the ECM-receptor interaction pathway had the most significant differences and the highest number of enriched factors, and the Pathways in cancer pathway had the highest number of genes.

### 3.5. Analysis of WNT pathway in hippocampus and PI3K-AKT pathway in PFC

**FIGURE 5:**
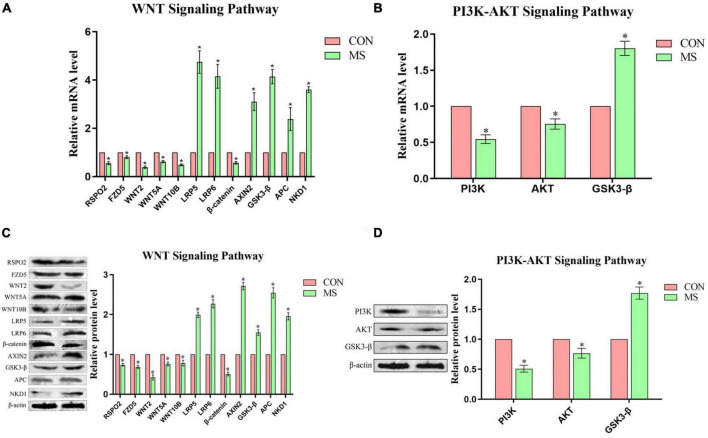
Effect of MS on the expression of genes related to the WNT signaling pathway in the hippocampal and PI3K-AKT signaling pathway in the PFC in piglets (*n* = 6). **(A)** Hippocampal WNT signaling pathway mRNA levels. **(B)** PFC PI3K-AKT signaling pathway mRNA levels. **(C)** Hippocampal WNT signaling pathway protein expression levels. **(D)** PFC PI3K-AKT signaling pathway protein expression levels. *P < 0.05.

By reviewing the literature and analyzing the results of previous transcriptomic analysis (KEGG), the experiment selected the WNT pathway related to neurodevelopment in the hippocampus and the PI3K-AKT pathway related to cognitive function in the PFC for research, the results were shown in Figure 5. In hippocampal WNT pathway-related genes, compared with the control group, the expression levels of RSPO2, FZD5, WNT2, WNT5A, WNT10B, and β-catenin were significantly down-regulated, while AXIN2, GSK3-β, APC, and LRP5/6 were significantly up-regulated in the MS group. In the PFC, MS treatment significantly inhibited the expression of PI3K/AKT/GSK3-β signal pathway.

## 4. Discussion

Maternal separation is extremely common in livestock production as an outcome of artificial nursing, or split-suckling. It is generally accepted that when experienced for a longer period of time intermittent neonatal MS can represent severe environmental deprivation (Gutman and Nemeroff, 2003). The MS model is recognized as the classic animal model for studying early-life stress, and early-life stress can severely affect the growth and development of the individuals. The effects of MS on brain development and function in rodents (Nishi et al., 2014) and primates (Levine and Mody, 2003) have been widely reported. As a biomedical model, pigs have the advantage of being highly similar to humans in many aspects, and this study will provide insights to better evaluate the mechanisms of stress effects on the offspring’s brain development and function during neonatal MS. High-throughput sequencing of the transcriptome, as a novel and efficient technology, has become an effective tool to assess the mechanisms of stress effects on the organism at the level of RNA (Peña et al., 2019). In this study, 1,632 DEGs in the hippocampus (1,077 upregulated and 555 downregulated) and 500 DEGs in the PFC (349 upregulated and 151 downregulated) were identified. qRT-PCR analysis of 9 genes in each the hippocampus and PFC showed expression trends consistent with RNA-seq, indicating that the RNA-seq data were reliable. Two pathways with significant differences in transcriptome analysis were also selected for the study, namely, the WNT signaling pathway in the hippocampus and the PI3K-Akt signaling pathway in the PFC. These results suggest that MS may cause changes in the transcriptional levels in the hippocampus and PFC of piglets and affect the development and cognitive function of the piglet brain.

Trauma occurs when animals are exposed to stresses in their early years that they cannot cope with, and these traumas are associated with declines in development of the PFC and hippocampus, which impedes overall development and functional expression of the organism’s brain. At the neurological level, early-life stress affects brain development, but how this stress affects brain development is still poorly understood, though neurons in hippocampal structures are known to be most sensitive to a noxious stimulus. In our experiment, transcriptome sequencing GO entries were screened for several genes associated with neurodevelopment, namely: protein phosphatase 3 catalytic subunit alpha (PPP3CA), ring finger protein 112 (RNF112), disabled homolog 1 (DAB1), neurogenic differentiation factor 1 (NEUROD1), and regulatory factor X3 (RFX3). PPP3CA encodes the calmodulin-binding catalytic subunit of calcineurin, which is closely related to development of the body. Some studies have found that PPP3CA knockout mice showed developmental defects in the nervous system (Qian et al., 2018). RNF112 is abundantly expressed in the brain and is regulated during brain development; mice with RNF112 knockout had poor brain development and showed impaired brain motor balance, spatial learning, and memory function (Tsou et al., 2017). DAB1 is critical in neuronal migration and formation in brain development, and is associated with neurodevelopmental disorders; accordingly, DAB1-deficient mice showed brain structural abnormalities, and model mice showing traits of autism had significantly reduced levels of DAB1 mRNA expression in the brain (Nawa et al., 2020). NEUROD1 is highly expressed in neurons, promoting neuronal differentiation and development during brain development. The expression of exogenous NEUROD1 activated the expression of transcription factors of astrocytes and promoted neuronal transdifferentiation (Ma et al., 2022), while NEUROD1 deficiency led to severe developmental defects in the brain (Naya et al., 1997). RFX3 is a regulator of cilia production in brain morphogenesis; in RFX3-deficient mice, dysplasia of the corpus callosum (the primary commissure connecting the cerebral hemispheres) and loss of the RFX3 function will lead to the distorted distribution of neurons (Bonnafe et al., 2004). Our experiment and the analyses of RNA-seq data showed that these genes related to neurodevelopment were downregulated by MS stress. These results are also consistent with the above research content. The expression level of the genes we screened should decrease in the case of neurodevelopmental dysplasia in the body, as demonstrated in our experiment with pigs. Therefore, from these results, it can be speculated that MS stress in piglets may inhibit the expression of these genes in the hippocampus, affecting neuron formation, development and differentiation, and thereby cognitive ability; nonetheless, the underlying mechanism requires further study. The hippocampus is a heterogeneous and complex region with different structures and regions playing different roles and responsible for different behavioral patterns (Fanselow and Dong, 2010). However, the whole hippocampal tissue was selected for this experiment, and there was no precise localization to the specific region of the hippocampus.

In this experiment, 9 differential genes were screened out through transcriptome sequencing GO entries: MC4R, NMB, GRP, ADCYAP1, CYFIP2, MAG, CPEB1, CPEB4, PLP1, these genes are closely related to cognitive memory. The melanocortin receptor (MCR) in the brain is related to cognitive function, which can improve the learning and memory ability of animals. Studies have found that neuroinflammation can reduce melanocortin system (MC4R and α-melanocyte-stimulating hormone) expression (Flores-Bastías et al., 2020). Neuromedin B (NMB) exerts its function through the NMB receptor. Stress can affect NMB receptor deficiency and impair memory (Yamada et al., 2003), suggesting that NMB and its receptors closely influence memory. Gastrin-releasing peptide (GRP) and its receptors are widely distributed in various brain regions, and GRP plays a key role in synaptic power, which is critical for cognitive function (Yang et al., 2017). Adenylate cyclase activating polypeptide 1 (ADCYAP1) acts as a neurotransmitter or neuromodulator that promotes neuronal survival. ADCYAP1 knockout mice show cognitive impairment in the novel object recognition test (Takuma et al., 2014). Cytoplasmic FMR1-interacting protein 2 (CYFIP2) plays a critical role in regulating actin dynamics and neuronal excitability in primary neurons in the medial PFC (mPFC). CYFIP2 protein expression is significantly reduced in Alzheimer’s disease cases, and cognitive behavioral tests showed that reduced CYFIP2 expression prevents mice from being able to solve the water maze (Tiwari et al., 2016) by affecting body spatial memory function. This study was consistent with the above gene expression changes, and it has been verified in pigs, indicating that the down-regulation of the expression levels of MC4R, NMB, ADCYAP1, GRP, and CYFIP2 is closely related to the cognitive impairment of piglets caused by MS stress. After brain injury, the myelin-associated glycoprotein (MAG), which inhibits axon growth, increases immunity. The findings have been reported in rodents where MAG was significantly increased in poison-stressed mouse brain tissue (Li et al., 2015). In this study, the brain injury of piglets after MS may be due to the inhibition of axonal growth due to the upregulation of MAG, resulting in impaired cognition. Cytoplasmic polyadenylation element-binding protein (CPEB) exists in neurons and glial cells. CPEB affects learning and memory, and affects neuronal morphology and synaptic plasticity, is a key mediator of important biological processes such as learning and memory. CPEB4 has a certain inhibitory effect on the development of brain nerves (Shin et al., 2016), but knockdown of CPEB1 expression inhibits the activation of astrocytes (Kim et al., 2011). In this study, we found that MS caused the down-regulation of CPEB1 and up-regulation of CPEB4 in the PFC, which affected the development of neurons, thereby impairing the cognitive function of piglets. Proteolipid protein 1 (PLP1) is a leading candidate gene for brain white matter structure and is essential for cognition and behavior. Prior studies have found that PLP1 overexpression can lead to progressive leukodystrophy and cognitive decline (Li et al., 2019). This proves that the PLP1 gene was up-regulated in piglets that experienced intermittent neonatal MS in this experiment, resulting in leukodystrophy and affecting cognitive function. The specific regulatory mechanism still needs to be further verified by methods or technologies.

The WNT signaling pathway is an evolutionarily conserved pathway that guides animal development and plays a key role in early development; it regulates the body’s neurogenesis, neuron differentiation, proliferation and migration, synaptogenesis and axon growth (Toledo et al., 2008). In addition, the WNT signaling pathway regulates neuronal maturation processes that depend on synaptic activity, and synaptic plasticity is closely related to cognitive ability. When the WNT signaling pathway is activated, the cognitive function will be improved and even cognitive deficits can be reversed (Vargas et al., 2014). Studies have found that WNT2 and WNT5a in the hippocampus may be related to nerve regeneration and are highly expressed (Sousa et al., 2010; Andersson et al., 2013). RSPO2 can activate the WNT signaling pathway (Zhang et al., 2019), and FZD5 can activate the WNT2, WNT10B and WNT5A proteins, but specifically interacts with WNt5A to induce the WNT signaling pathway (Ishikawa et al., 2001). WNT proteins interact with the Frizzled (Fz)/low-density lipoprotein (LDL) LRP complex on the cell surface of target cells. And signal transduction to dishevelned (Dsh), GSK-3β, axin, adenomatous polyposis coli (APC), and the transcriptional activity of β-catenin are regulated by the GSK3β/APC/axin complex. When cells receive a WNT signal, the complex is inhibited, and β-catenin increases in the cytoplasm and nucleus to participate in the regulation of target gene transcription (Logan and Nusse, 2004). NKD1 can inhibit the WNT signaling pathway by preventing the nuclear accumulation of β-catenin (Angonin and Van Raay, 2013). Fatima et al. (2019) established that chronic unpredictable stress induces upregulation of GSK3β in the WNT signaling pathway in the neonatal brain, and inhibits β-catenin expression, which in turn impairs neurogenesis and affects brain development. This is consistent with our findings. Similarly, it has been concluded that early-life stress leads to neural differentiation and proliferation defects in the offspring due to inhibition of the WNT signaling pathway (Yde et al., 2020). Li et al. (2022) found that stress induced by toxic substances caused oxidative stress in zebrafish, downregulated the expression of genes related to the WNT signaling pathway and interfered with neural development, while treatment with WNT activators rescued the toxic response. The present results are consistent with the above findings that external adverse stimuli can affect the neurodevelopment of the organism. Our study also showed that when piglets were exposed to adverse external stimuli, the neurodevelopment of the hippocampus was affected because of inhibition of the WNT signaling pathway, which consequently diminished the cognitive ability of the piglets.

The PI3K/AKT pathway is an intracellular signaling pathway closely related to extracellular signaling, promoting the metabolism of cell growth and proliferation, cell survival, and angiogenesis. Numerous studies have demonstrated that the activation of PI3K and AKT can reduce neuronal damage and promote neuronal activation. The tropomyosin-related kinase B (TrkB) pathway protects against traumatic brain injury by activating the PI3K/AKT pathway, which can cause cognitive deficits to some extent, and blocking AKT activation with specific PI3K inhibitors eliminates the protection against brain tissue damage (Wu et al., 2014). Activation of the PI3K/AKT pathway has also been found to significantly reduce neuronal damage in ischemic stroke (Lo et al., 2003), as AKT phosphorylates its downstream target GSK-3β, and the PI3K/AKT/GSK3β pathway is essential for maintaining cell survival. Activation of GSK3β was found to occur in cognitively deficient cell models in *in vitro* experiments (Alvarez et al., 2001). It has also been demonstrated that inhibition of GSK3β can reduce the accumulation of Aβ in the body to a certain extent and can alleviate the reduction of spatial learning and memory in transgenic animals with Alzheimer’s disease (Ding et al., 2008). Zheng et al. (2017) concluded that the naturally occurring amino acid selenomethionine increased AKT and PI3K activity in neurons, significantly inhibited GSK3β activity in the brains of mice with Alzheimer’s, and enhanced the proliferation and differentiation of neural stem cells by activating the PI3K-AKT-GSK3β-related pathway. The PI3K-AKT pathway also plays a crucial role in neuronal development in the brain and is instructive for many important processes related to brain development. When the PI3K-AKT-GSK3β pathway is activated in mice modeling neurodevelopmental disorders, it protects against neurodevelopmental (Wang et al., 2022). The experimental results reported in the above studies are consistent with the present results using pigs as the experimental subject, whereby MS stress caused inhibition of the PI3K-AKT-GSK3β pathway in the PFC of piglets, which thereby impaired the cognitive ability of the piglets.

## 5. Conclusion

In summary, intermittent neonatal maternal separation leads to changes in gene expression levels in the hippocampus and PFC of piglets, thereby impairing neurodevelopment and differentiation in the piglet brain.

## Data availability statement

The datasets presented in this study can be found in online repositories. The names of the repository/repositories and accession number(s) can be found in this article/Supplementary material.

## Ethics statement

The animal studies were approved by the Animal Protection and Utilization Committee of Northeast Agricultural University (NEAUEC20200346). The studies were conducted in accordance with the local legislation and institutional requirements. Written informed consent was obtained from the owners for the participation of their animals in this study.

## Author contributions

HL, WZ, and SZ: conceived and designed the current experiments plans. SZ: wrote the manuscript and completed the experiments. YY: performed the experiments. ZC: analyzed these data. MW: provided the help during animal breeding. QH: resources and supervision. WZ and HL: revised the whole manuscript. All authors contributed to the article and approved the submitted version.
