# Supplementary material for: Effects of early maternal separation on the expression levels of hippocampal and prefrontal cortex genes and pathways in lactating piglets
Source: Front Mol Neurosci. 2023 Aug 14;16:1243296. doi: 10.3389/fnmol.2023.1243296 (PMC10460909; doi:10.3389/fnmol.2023.1243296)
Supplement: Supplementary file 1 [file Table_1.docx]

Supplementary Material

**Effects of early maternal separation on the expression levels of hippocampal and prefrontal cortex genes and pathways in lactating piglets**

**Sitong Zhou^1†^, Yue Yang^1†^, Zheng Cheng^a^, Mengyao Wu^a^, Qi Han^a^, Wenzhong Zhao^b*^, Honggui, Liu^ac*^**

*** Correspondence:**

Corresponding author: Honggui Liu. Email: [liuhonggui1312@163.com,](mailto:liuhonggui1312@163.com,) Wenzhong Zhao. Email: wzzhao@neau.edu.cn

**1 Supplementary Tables**

**Table S1 The compositions of sows and piglets feed**

| Basic diet | Content | |
| --- | --- | --- |
|  | Lactating sows feed | Piglets creep feed |
| Crude protein | ≥ 17.0% | ≥ 18.0% |
| Crude fiber | ≤ 7.0% | ≤ 4.0% |
| Coarse ash | ≤ 9.0% | ≤ 7.0% |
| Moisture | ≤ 14.0% | ≤ 14.0% |
| Total phosphorus | ≥ 0.5% | ≥ 0.5% |
| Sodium chloride | 0.3-1.2% | 0.3-1.49% |
| Calcium | 0.7-1.3% | 0.4-1.5% |
| Lysine | ≥ 1.1% | ≥ 1.35% |

% represents the proportion of a nutrient content in the total feed, the feed is solid feed.

**Table S2 The top 25 GO entries with the most significant enrichment in the hippocampal and prefrontal cortex**

| GO term | |
| --- | --- |
| hippocampal | prefrontal cortex |
| signal transduction | positive regulation of transcription by RNA polymerase II |
| positive regulation of transcription by RNA polymerase Ⅱ | regulation of transcription , DNA - templated |
| regulation of transcription , DNA - templated | negative regulation of transcription by RNA polymerase Ⅱ |
| protein phosphorylation | signal transduction |
| positive regulation of transcription , DNA - templated | cell adhesion |
| negative regulation of transcription by RNA polymerase Ⅱ | positive regulation of cell population proliferation |
| G protein - coupled receptor signaling pathway | protein phosphorylation |
| transmembrane transport | positive regulation of transcription , DNA - templated |
| oxidation - reduction process | transmembrane transport |
| proteolysis | proteolysis |
| intracellular signal transduction | oxidation - reduction process |
| cell adhesion | G protein -- coupled receptor signaling pathway |
| ion transport | regulation of signaling receptor activity |
| phosphorylation | positive regulation of gene expression |
| positive regulation of gene expression | immune response |
| negative regulation of transcription , DNA - templated | angiogenesis |
| positive regulation of GTPase activity | extracellular matrix organization |
| positive regulation of cell population proliferation | lipid metabolic process |
| negative regulation of cell population proliferation | microtubule - based movement |
| regulation of transcription by RNA polymerase Ⅱ | positive regulation of cell migration |
| cell migration | regulation of transcription by RNA polymerase Ⅱ |
| protein ubiquitination | defense response to virus |
| regulation of signaling receptor activity | cell migration |
| positive regulation of cell migration | ion transport |
| negative regulation of apoptotic process | positive regulation of angiogenesis |
| membrane | membrane |
| integral component of membrane | integral component of membrane |
| cytoplasm | nucleus |
| plasma membrane | cytoplasm |
| nucleus | plasma membrane |
| cytosol | cytosol |
| nucleoplasm | extracellular space |
| extracellular space | nucleoplasm |
| integral component of plasma membrane | extracellular region |
| extracellular region | integral component of plasma membrane |
| Golgi apparatus | endoplasmic reticulum |
| endoplasmic reticulum | cell surface |
| intracellular membrane - bounded organelle | collagen - containing extracellular matrix |
| cell surface | extracellular matrix |
| mitochondrion | mitochondrion |
| protein binding | protein binding |
| metal ion binding | metal ion binding |
| ATP binding | nucleotide binding |
| nucleotide binding | ATP binding |
| transferase activity | calcium ion binding |
| DNA binding | DNA binding |
| calcium ion binding | zinc ion binding |
| identical protein binding | DNA - binding transcription activity |
| hydrolase activity | nucleic acid binding |
| protein kinase activity | protein homodimerization activity |
